# Early and rapid diagnosis of *Chlamydia psittaci* pneumonia by tNGS in six patients: a case series

**DOI:** 10.3389/fmed.2024.1491838

**Published:** 2024-11-27

**Authors:** Xinsheng Yan, Huali Fu, Wenjun Deng, Zhenlu Zhang, Dong Wang

**Affiliations:** ^1^Department of Clinical Laboratory, Wuhan Asia General Hospital, Wuhan Asia General Hospital Affiliated to Wuhan University of Science and Technology, Wuhan, China; ^2^Respiratory and Critical Care Medicine, Wuhan Asia General Hospital, Wuhan Asia General Hospital Affiliated to Wuhan University of Science and Technology, Wuhan, China; ^3^Department of Radiology, Wuhan Asia General Hospital, Wuhan Asia General Hospital Affiliated to Wuhan University of Science and Technology, Wuhan, China

**Keywords:** psittacosis, *Chlamydia psittaci*, target next-generation sequencing, tNGS, pathogen detection

## Abstract

**Background:**

Psittacosis is a zoonotic infectious disease caused by *Chlamydia psittaci* (*C. psittaci*) infection, which can be transmitted by birds, poultry and wild animals. The symptoms and imaging findings of *C. psittaci* pneumonia are atypical and primarily rely on etiological diagnosis. The incidence of *C. psittaci* infection has been significantly underestimated because of the low sensitivity and poor timeliness of traditional diagnostic methods. Therefore, early and accurate diagnosis of psittacosis remains a challenge.

**Case presentation:**

A case series with six pneumonia patients who were admitted to our hospital in the period from January 2023 to June 2023 is presented. These patients exhibited acute onset and symptoms, including fever, cough, poor appetite, dry mouth, dizziness, chills, and chest tightness. Despite comprehensive laboratory and radiological examinations, the cause of the pneumonia remained unidentified. Therefore, a sample of bronchoalveolar lavage fluid (BALF) was tested via target next-generation sequencing (tNGS), which revealed a positive result for *C. psittaci*. Prompt adjustment of the treatment regimens upon identification of the pathogen led to favorable outcomes in all patients.

**Conclusion:**

tNGS is a novel diagnostic technology that enables rapid, accurate and cost-effective detection of *C. psittaci* pneumonia. Early detection of *C. psittaci* can improve patient outcomes through timely adjustment of therapies.

## 1 Introduction

According to the Global Burden of Disease Collaboration, it is estimated that nearly 600 million individuals were estimated to be affected by pneumonia and other lower respiratory tract infections globally in 2019, resulting in 2.5 million deaths (1). The epidemiologic distribution of pneumonia pathogens has shifted in recent years, with an increase in some rare pathogens. *C. psittaci*, an intracellular gram-negative pathogen, is commonly found in birds (especially parrots and pigeons) and mammals (2). Inhalation of aerosols from infected bird feathers or avian excreta can lead to *C. psittaci* pneumonia in humans. This pathogen is frequently misdiagnosed, leading to the potential misuse of antimicrobials and the development of drug resistance (3, 4). Patients with *C. psittaci* pneumonia typically exhibit sepsis and a rapidly deteriorating condition. Early diagnosis and treatment are crucial for improving patient prognosis because of the rapid progression of the disease (5).

The diagnosis of *C. psittaci* pneumonia is challenging because of its nonspecific clinical presentation and often relies on laboratory testing for the pathogen. Currently, culture, serology and molecular testing are the main methods of detection. Cell cultures for *C. psittaci* are time-consuming and require a high level of laboratory biosafety, making them unsuitable for routine use, and serologic tests have limited early diagnostic value, as they are more appropriate for retrospective diagnosis (6). PCR reagents for *C. psittaci* are not readily available and are limited by clinician judgment, although PCR is considered the gold standard for detection (7). Metagenomic next-generation sequencing (mNGS) employs high-throughput sequencing technology to capture the comprehensive range of microbial nucleic acid sequences present in samples. These sequences are then compared and analyzed with existing microbial nucleic acid sequences stored in a database. This approach facilitates the efficient and accurate identification of suspected pathogenic microorganisms in samples. In recent years, many reported cases of *C. psittaci* pneumonia have been definitively diagnosed by mNGS (8, 9). More recently, target next-generation sequencing (tNGS), a new technology based on the combination of ultra-multiplex PCR amplification and high-throughput sequencing, has gradually entered clinic practice as a faster and less expensive molecular detection method than mNGS. However, reports on the application of tNGS for *C. psittaci* detection are scarce. Therefore, this study was conducted to analyze the clinical data of six patients with *C. psittaci* pneumonia at our hospital to evaluate the effectiveness of tNGS for early and rapid diagnosis and to better understand the clinical characteristics of these patients.

## 2 Case presentation

Between January 2023 and June 2023, a total of 297 inpatients underwent tNGS testing, and six of these patients were diagnosed with *C. psittaci* pneumonia. Data regarding the demographic characteristics of the six patients, including age, gender, occupational history, and avian exposure, were collected. Clinical manifestations, signs, as well as laboratory and imaging findings, were documented. The aetiological diagnosis was based on the sequence of the *C. psittaci* obtained from BALF samples via tNGS. Furthermore, the use of antibiotics prior to and following the diagnosis of *C. psittaci* pneumonia, along with the time intervals from symptom onset to admission and from admission to diagnosis, were recorded.

### 2.1 Baseline characteristics and clinical manifestations

Table 1 presents general information on the six patients, comprising three men and three women aged between 18 and 66 years, who were included in this study. Among them, two patients had a history of hypertension, three had a history of diabetes, one had a history of atrial fibrillation, one had a history of ulcers, and one had abnormal liver function. Additionally, three patients had a documented history of exposure to poultry, whereas two had a history of exposure to parrots. All patients experienced fever, with the highest body temperature recorded ranging from 38.0 to 40.0°C. Common accompanying symptoms included cough (3/6), headache (2/6), dizziness (2/6), cold symptoms (2/6), stiffness (3/6), chest tightness (2/6), pharyngeal pain (1/6), fatigue (4/6), muscle pain (2/6), and frequent urination urgency (2/6).

**Table 1:** Baseline characteristics and clinical manifestations of patients with *Chlamydia psittaci* pneumonia.

Case	Sex	Age (year)	Occupation	Past history	Contact history	Temperature peak (°C)	Initial symptoms	Concomitant symptoms
1	Male	63	Retired	Diabetes	No	38	Fever, headache, fear of the cold	Chest tightness and shortness of breath
2	Male	48	Company employee	Diabetes; Hypertension; Atrial fibrillation	No	40	Fever, giddy	Fatigue, poor appetite, cough
3	Female	66	Retired	Atherosclerosis of the aorta; Liver dysfunction	No	39	Fever, Fear of the cold	Fever, dry cough, fatigue, dysuria, cough
4	Female	37	Company employee	Diabetes; Hypertension	Parrot	38.5	Fever, chest distress	Cough, sore throat, headache, muscle aches
5	Female	58	Unemployed	Gastric ulcer; duodenal ulcer	Chicken	40	Fever	Muscle aches, dizziness, fatigue
6	Male	18	Student	No	Parrot	39.5	Fever	Weakness, poor stamina

### 2.2 Clinical laboratory testing

Various parameters, including the white blood cell (WBC) count, neutrophil (NE) percentage, ultrasensitive c-reactive protein (hCRP) level, serum amyloid A (SAA) level, procalcitonin (PCT) level, erythrocyte sedimentation rate (ESR), alanine aminotransferase (ALT) level, aspartate aminotransferase (AST) level, glomerular filtration rate (eGFR) and interleukin 6 (IL-6) level, were assessed upon admission (Table 2). All six patients presented normal WBC counts, with three patients showing elevated NE percentages. Furthermore, all patients presented increased levels of hCRP, SAA, PCT, and IL-6. During the initial visit, the recorded levels ranged from 33.87–187.20 mg/L, 266.64–498.99 mg/L, 0.054–3.854 ng/mL and 26.90–61.90 pg./L, respectively. The ESR ranged from 13 to 88 mm/h, with increases noted in 4 of 6 patients. ALT levels ranged from 18 to 61 U/L, with elevated levels observed in 2 of the 6 patients. AST levels fell within the range of 16–50 U/L, and elevated levels were observed in 1 of the 6 patients. Additionally, the eGFRs ranged from 63 to 124 mL/min, with elevated levels noted in 5 of the 6 patients.

**Table 2:** Laboratory results of the patients with *C. psittaci* pneumonia.

Case	WBC	NE	hCRP	SAA	PCT	ESR	ALT	AST	eGFR	IL-6
	(*10^9/L)	(%)	(mg/L)	(mg/L)	(ng/mL)	(mm/h)	(U/L)	(U/L)	(mL/min)	(pg/mL)
1	8.82	75.4	187.20	450.34	3.854	88	34	31	63	61.90
2	5.74	89.4	116.55	352.11	0.127	19	21	24	110	50.65
3	5.10	70.4	33.87	339.28	0.074	45	61	50	92	33.50
4	9.48	71.7	59.76	498.99	0.054	53	43	16	124	26.90
5	7.26	74.1	61.93	266.64	0.065	49	12	19	97	57.20
6	7.52	80.0	100.85	431.63	0.424	13	18	26	109	55.60
Ave	7.32	76.8	93.36	389.83	0.766	45	32	28	99	47.63
NR	3.5–9.5	40–75	0–3	0–10.8	0–0.046	0–26	7–40	16–50	66–143	0–7

Ave, average; NR, normal range.

### 2.3 Imaging findings

**Figure 1:**
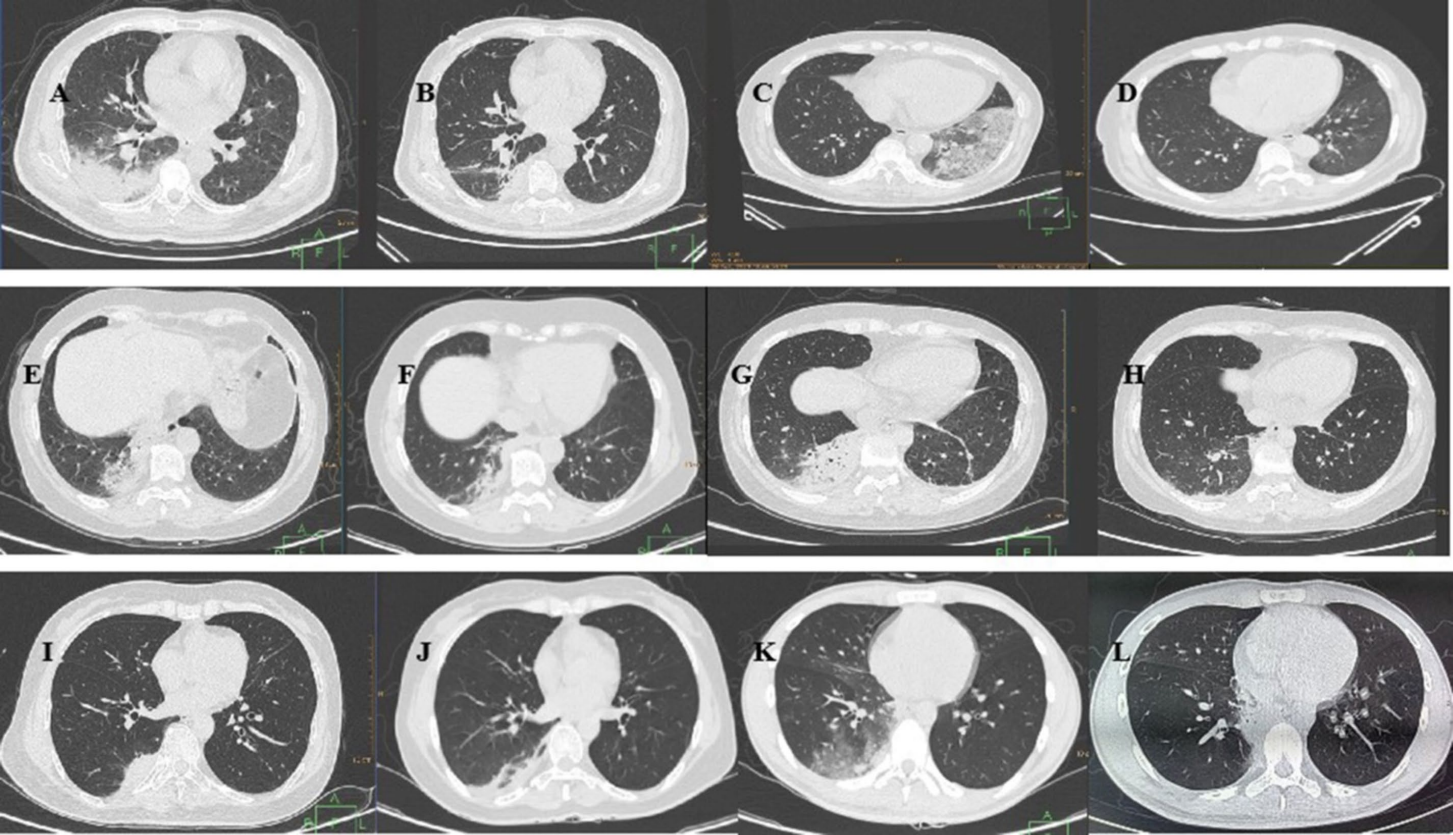
Chest CT of the six patients with *Chlamydia psittaci* pneumonia. Case 1: (A) On January 30, the lower lobe of the right lung had a large area of consolidation, and the ventilation bronchus was visible. (B) On February 10, shadow absorption in the lower lobe of the right lung was significantly reduced. Case 2: (C) On February 9, a chest CT revealed diffusely distributed ground–glass shadows with patchy solid shadows in the lower lobe of the left lung. (D) On February 28, a small number of pale shadows were observed in the lower lobe of the left lung, and the foci of intrapulmonary infection were mostly resorbed. Case 3: (E) On March 11, a solid lesion was detected in the posterior basal segment of the lower lobe of the right lung. (F) On March 18, there was a decreased lesional area and partial thinning of the posterior basal segment of the lower lobe of the right lung. Case 4: (G) On April 10, a chest CT revealed a large solid lesion in the lower lobe of the right lung and a striated shadow in the lower lobe of the left lung. (H) On April 17, chest CT revealed that the solid lesion in the original lung was largely absorbed, as was the striated shadow in the left lower lobe. Case 5: (I) On April 12, a chest CT revealed a solid shadow in the posterior basal segment of the lower lobe of the right lung. (J) On April 21, a chest CT revealed inflammatory thinning of the lower lobe of the right lung. Case 6: (K) On June 26, there were multiple ground glass shadows in the middle lobe and lower lobe of the right lung, and patchy consolidation was observed in the lower lobe of the right lung. (L) On July 8, a chest CT revealed that the inflammation in the lower lobe of the right lung had resolved.

Chest CT was conducted for all patients upon admission (Figure 1). Among the six patients, five had lesions affecting one lung, whereas one patient had bilateral lung involvement. The lesions were diffusely distributed ground-glass shadows with localized solid lesions, and two patients presented bronchial air signs.

### 2.4 Targeted next-generation sequencing

**Figure 2:**
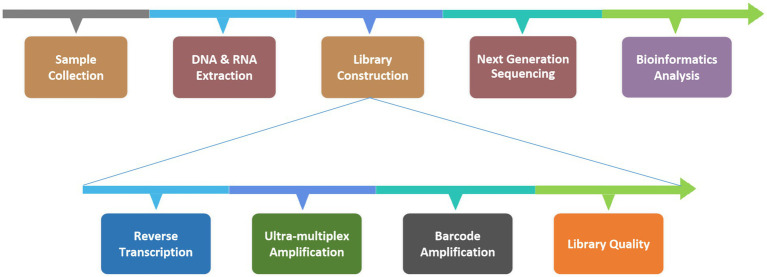
Protocol for the tNGS assay.

Six patients with suspected *C. psittaci* pneumonia underwent bronchoscopy to obtain BALF samples which were then sent to the KingMed Diagnostics Group Co., Ltd., Guangzhou, China for tNGS. *C. psittaci* was detected in all six cases, other pathogens were identified in two of the cases (Table 3). The protocol for the tNGS assay is showed in Figure 2. After the samples were collected in the clinical laboratory, nucleic acids (DNA and RNA) were extracted, after which the tNGS target library was constructed and sequenced. The target 198 pathogens (Supplementary Table S1) of the tNGS assay include 80 bacteria, 79 viruses, 32 fungi and 7 atypical pathogens. Three amplicons (Supplementary Table S2) were designed for the detection of *C. psittaci*.

**Table 3:** Diagnosis and treatment of patients with *C. psittaci* pneumonia.

Case	Anti-infective drugs used before diagnosis	Time from symptom onset to admission	Time from admission to diagnosis	Turnaround time of tNGS from sample receipt to final report	tNGS results (RPhK)	Anti-infective drugs used after diagnosis
1	Ceftazidime, Piperacillin/Tazobactam, Moxifloxacin	7 d	4 d	12 h	*C. psittaci* 757	Moxifloxacin
2	Ceftazidime, Cefoperazone/Sulbactam, Moxifloxacin	7 d	4 d	18 h	*C. psittaci* 2,718, *Staphylococcus aureus* 90	Moxifloxacin, Minocycline
3	Piperacillin/Tazobactam, Fosfomycin	5 d	4 d	21 h	*C. psittaci* 1735, *Cryptococcus neoformans* 97	Levofloxacin
4	Penicillin, Cefaclor	8 d	2 d	17 h	*C. psittaci* 22	Moxifloxacin
5	Cefminox, Biapenem, Minocycline	4 d	4 d	19 h	*C. psittaci* 8,048	Moxifloxacin, Minocycline
6	Cefpodoxime, Abidor, Etimicin, Amoxicillin/Clavulanic acid	4d	2d	16 h	*C. psittaci* 9,652	Moxifloxacin, Minocycline

#### 2.4.1 Material processing and nucleic acid extraction

BALF samples were collected according to the established standard procedures. For viscous BALF samples, an equal volume of 0.1 mol/L dithiothreitol (DTT) liquefaction agent was added to the collection tube; the mixture was then vortexed thoroughly and incubated at room temperature for 3–5 min to ensure complete liquefaction. Non-viscous BALF samples did not require a 0.1 mol/L DTT treatment step. A total of 13 μL of exogenous endogenous reference was added to 1.3 mL of the liquefied mix; the mixture was vortexed to ensure thorough mixing and then centrifuged at 12,000 rpm for 5 min. The supernatant was discarded, and the residual sample volume was adjusted to 500 μL by pipetting. This aliquot was transferred into the bead mill tube provided in the extraction kit, to which 50 μL of SDS was added, and the mixture was subjected to a wall-breaking apparatus for mechanical lysis. Following mechanical lysis, the samples were centrifuged at 12,000 rpm for 5 min, and 250 μL of the supernatant was used for nucleic acid extraction. The extraction was performed via the MagPure Pathogen RNA/DNA Extraction Kit (Magen Biotechnology, Guangzhou, China), following the manufacturer’s protocol. The extracted nucleic acids were quantified via an Equalbit DNA HS Assay Kit (Vazyme Biotech, Nanjing, China) with an Invitrogen™ Qubit™ 4.0 (Thermo Fisher Scientific, Massachusetts, United States), and the input nucleic acids did not exceed 100 ng for library construction.

#### 2.4.2 Library preparation and sequencing

Library preparation was performed via the RP100TM Respiratory Pathogen Microorganisms Multiplex Testing Kit (KingCreate Biotechnology, Guangzhou, China). cDNA was synthesized via reverse transcription of the extracted nucleic acids, followed by steps such as ultra-multiplex PCR amplification, PCR product purification, adapter ligation and library purification to complete library construction. The constructed libraries were pooled into homogeneous masses. The size of the library fragments was determined via the Qsep100 Bio-Fragment Analyzer (BiOptic, Taiwan, China). The size of the library fragments should be of from 250 to 350 bp. The qualified pooled library was diluted and denatured, 500 μL of which was subjected to the Illumina MiniSeq Platform (Illumina, California, United States) for sequencing.

#### 2.4.3 Bioinformatics analysis

The raw sequencing read data were subjected to a quality control procedure. Fastp v0.20.1 was employed for adapter trimming and quality trimming using default parameters, followed by mapping to the reference via Bowtie2 v2.4.1 in ‘very-sensitive’ mode. The reference sequence used for read mapping was a database curated from various sources including the GenBank, RefSeq, and Nucleotide databases from NCBI.^1^ To identify positive signals for specific pathogens, the number of mapped reads was counted and normalized to the number of reads per 100,000 (RPhK). Cases with specific RPhKs were considered positive for each sample.

#### 2.4.4 Interpretation of tNGS results

For bacteria (excluding the *Mycobacterium tuberculosis* complex), fungi, viruses, and atypical pathogens, the criteria for positivity are as follows: (1) RPhK ≥10 when amplicon coverage is 100% and the number of amplicons is greater than 1; (2) RPhK ≥30 when 50% ≤ amplicon coverage <100% and the number of amplicons is equal to 1; (3) RPhK ≥50 when amplicon coverage is less than 50%. For *Mycobacterium tuberculosis* complex: a positive result is considered when RPhK ≥1, no other samples in the same batch detect *Mycobacterium tuberculosis*, and the retest result is ≥1. Additionally, the detected pathogens are classified according to their pathogenicity: (A) specifically pathogenic in respiratory specimens or clinically common pathogens; (B) opportunistic (conditional) pathogens in respiratory specimens that may cause infection in patients with systemic or local immunocompromise/compromise/deficiency, respiratory barrier disruption, or lower respiratory microecological imbalance; (C) the normal microecological flora of the respiratory tract, usually does not lead to infection. Finally, we need to perform a thorough assessment of the patient’s clinical profile to determine the presence of a lung infection and the clinical relevance of the potential pathogen. This evaluation includes the patient’s medical history, symptoms, imaging findings, tNGS results and other laboratory findings.

### 2.5 Treatment and outcome

The diagnosis and treatment histories of the six patients with *C. psittaci* pneumonia are shown in Table 3. All patients received empiric anti-infective therapy with two to four antibiotics sequentially prior to diagnosis, with one patient also receiving antiviral therapy using abidor. The six patients experienced a mean time of 5.8 days from symptom onset to hospital admission (range 4–8 days) and a mean time of 3.3 days from hospital admission to diagnosis (range 2–4 days). For all samples, the tNGS turnaround time from sample receipt to the final report was less than 24 h. Additional pathogens were found in the tNGS results of two patients, but were subsequently excluded by other laboratory findings and clinical manifestations. Six patients were eventually diagnosed with *C. psittaci* pneumonia. Following diagnosis, the treatment regimen was promptly adjusted to quinolone antibiotic therapy for anti-infection purposes. After 1 week of anti-infection treatment, all six patients were discharged once their body temperature normalized and their clinical symptoms significantly improved. Prior to discharge, all patients underwent chest CT examinations, which revealed the disappearance of inflammatory lesions.

## 3 Discussion

Studies have indicated that the global prevalence of *C. psittaci* infection in birds is as high as to 20%, with all birds posing a potential risk for *C. psittaci* infection in humans (10). In the present study, three patients had confirmed contact with birds in their prehistory. This case report suggests that since close contact with birds is one of the major risk factors for infection with the pathogen, it is important to elicit the patient’s history in detail to provide direction for pathogen detection. All six patients in this case series experienced a fever between 38.0 and 40.0°C and symptoms such as cough, headache, sore throat, malaise and other flu-like symptoms. These signs and symptoms lack specificity and are similar to those of community-acquired pneumonia caused by various pathogens including viruses, fungi and bacteria. Owing to the diverse and atypical nature of the symptoms of psittacosis, its incidence has been significantly underestimated compared with that of other atypical pathogens (11). Our data further support previous reports on this matter.

Similarly, inflammatory biomarkers are nonspecific for *C. psittaci* pneumonia. The WBC count, PCT level, and ESR demonstrated limited diagnostic value for *C. psittaci* pneumonia in this study. hCRP, SAA, and IL-6 showed some diagnostic potential, but their elevation is not exclusive to this pathogen and lacks specificity. In summary, the laboratory findings in *C. psittaci* pneumonia are nonspecific, which aligns with the findings from previous studies (12). Chest CT scans of all patients revealed extensive lung lesions with varying degrees of pulmonary infiltrates. Unilateral lung involvement was common, but bilateral lesions were also observed, both of which were nonspecific. While *C. psittaci* infection is often linked to bird contact, it is not a prerequisite for diagnosis. Therefore, pathogen detection plays a crucial role in confirming the diagnosis of *C. psittaci* pneumonia (13).

Nonetheless, conventional methods for detecting pathogens such as culture or serological tests, are not suitable for early detection of the disease (14). Compared with traditional methods, mNGS, known for its high sensitivity and broad detection range, plays a crucial role in guiding the diagnosis and treatment of pneumonia (15). Previous studies have investigated the use of mNGS for the detection of *C. psittaci* infection, and this method has demonstrated high detection efficacy (8, 16–19). Moreover, mNGS has gained popularity for its ability to detect multiple pathogens simultaneously (20, 21). The rise in the diagnosis and reporting of psittacosis in recent years may be linked to the increased clinical utilization of mNGS (22). However, owing to its cost and long reporting time, mNGS may not be feasible in all cases and is typically used as a complementary option for challenging clinical scenarios. In China, the current price of mNGS in 2024 is US$ 500–700 per sample, whereas the price of tNGS is US$ 100–200. To control the cost of mNGS, batch processing of samples is often necessary, resulting in longer turnaround times. The tNGS test offers advantages such as fast detection speed, wide coverage and high accuracy, similar to those of the mNGS test, but incurs only a quarter of the cost (23). The time span from admission to diagnosis for the six patients in this study ranged from 2 to 4 days, which was notably shorter than the 5 to 11 days reported by Dai et al. for six patients diagnosed with *C. psittaci* pneumonia via mNGS (24). tNGS focuses on dozens to hundreds of clinically common and subrare pathogens, dramatically reducing the amount of sequencing data to 0.1 M through targeted enrichment technology, which in turn dramatically reduces the cost of testing (25). Furthermore, tNGS allows for dual-process DNA and RNA assays in a single experiment, is less impacted by prior antibiotic exposure, and excels in diagnosing rare, atypical pathogens while simultaneously identifying coinfecting pathogens. In this study, *C. psittaci* was consistently detected in all the BALF samples tested. Following prompt and targeted anti-infective treatment, the patients experienced a significant improvement in their symptoms, and imaging results revealed resolution of most of the infected foci. The use of tNGS technology in this study proved to be crucial in identifying the source of infection.

BALF is regarded as the most reliable specimen for the detection of pathogens in cases of lower respiratory tract infection (26, 27). However, BALF samples are challenging to obtain in some hospitals where bronchoscopy is unfeasible. Compared with BALF, sputum specimens are relatively easy to collect and have wider application and operability in practice. Zhenfeng Deng et al. (28) compared the detection of 209 sputum samples with tNGS and conventional microbiological testing methods, and found that the overall microbial detection rate of tNGS was significantly higher than that of conventional microbiological testing (96.7% vs. 36.8%). Their study suggests that tNGS may also be a valuable pathogenic diagnostic method for patients for whom alveolar lavage fluid collection is not appropriate.

In summary, the clinical manifestations of *C. psittaci* pneumonia can be complex, diverse and nonspecific. Clinicians should have a high index of suspicion for *C. psittaci* infection in patients with a history of live poultry exposure, who present with high fever and cough, along with significantly elevated levels of hCRP, SAA, and IL-6, slightly elevated PCT levels, normal WBC counts, and negative results of routine aetiological tests. Under the above circumstances, tNGS, as an emerging diagnostic technique for pathogenic microorganisms, is expected to be an economical, rapid and accurate method for diagnosing *C. psittaci* pneumonia.

## Data Availability

The original contributions presented in the study are included in the article/Supplementary material, further inquiries can be directed to the corresponding author.

## Footnotes

^1^ https://www.ncbi.nlm.nih.gov
